# Open-wedge high tibial osteotomy for spontaneous osteonecrosis of the medial tibial plateau shows excellent clinical outcomes

**DOI:** 10.1186/s40634-020-00231-z

**Published:** 2020-03-14

**Authors:** Kenichi Goshima, Takeshi Sawaguchi, Kenji Shigemoto, Shintaro Iwai, Kenji Fujita, Yuki Yamamuro

**Affiliations:** Department of Orthopedic Surgery and Joint Reconstructive Surgery, Toyama Municipal Hospital, 2-1 Imaizumi Hokubu-machi, Toyama, 939-8511 Japan

**Keywords:** Open-wedge high tibial osteotomy, osteonecrosis, medial tibial plateau

## Abstract

PurposeThere have been few reports on medial tibial plateau osteonecrosis, and treatment options remain controversial. This study aimed to evaluate the clinical outcomes of open-wedge high tibial osteotomy (OWHTO) for osteonecrosis of the medal tibial plateau.MethodsPatients who underwent OWHTO for spontaneous osteonecrosis of the medial tibial plateau from November 2013 to September 2017 at our institution and followed up for at least 2 years after surgery were included in this study. Patients with history of alcohol abuse and corticosteroid therapy were excluded. Clinical evaluations, including the Japanese Orthopedic Association (JOA) score and the Oxford Knee Score (OKS), were measured preoperatively and at the final followup. Radiological evaluations included the weight-bearing line ratio (WBLR) and the lesion stage of the osteonecrosis according to Carpintero, Lotke, and the modified Ficat and Arlet classification. The area and size of the necrosis and the type of meniscus tear were also evaluated using preoperative magnetic resonance imaging (MRI). Additionally, cartilage regeneration was evaluated at plate removal.ResultsTwelve cases that underwent OWHTO for spontaneous osteonecrosis of the medial tibial plateau were enrolled. Eleven cases had isolated medial tibial osteonecrosis, and one case had both femoral and tibial osteonecrosis. The mean age was 59.6 ± 9.0 years, and the mean follow-up period was 41.8 ± 17.6 months.The WBLR significantly changed after OWHTO (24.0% ± 10.7% to 66.3% ± 6.7%, *P* < 0.001), and all clinical scores significantly improved after surgery: JOA score 63.3 ±12.3 to 95.0 ± 4.8, OKS 27.4 ± 7.8 to 42.6 ± 4.1, both 0.001. There were no adverse complications requiring additional surgery. The MRI findings revealed that all cases had meniscal lesions in addition to a necrotic lesion. Second-look arthroscopy was performed at plate removal in 11 cases, and cartilage regeneration was observed in 9/11 cases (81.8%).ConclusionsThis study’s results demonstrated that OWHTO is an effective procedure for spontaneous osteonecrosis of the medial tibial plateau with respect to subjective and objective clinical outcomes.

## Introduction

Spontaneous osteonecrosis of the knee (SONK) in the medial tibial plateau was first reported by D’Anglejan et al. in 1976 [1]; this is a rare condition compared to SONK in the medial femoral condyle, and it represents only 2% of all osteonecrosis cases reported in the knee [2–5]. Similar to SONK in the femoral condyle, most patients with osteonecrosis of the medial tibial plateau are women older than 60 years who have sudden pain onset on the medial side of the knee, which is often related to minor trauma or an increase in activity [6].

Treatment of osteonecrosis of the medial tibial plateau remains controversial due to its rarity, and various options such as conservative non-weight-bearing treatment, arthroscopic drilling, osteochondral graft, high tibial osteotomy (HTO), unicompartmental knee arthroplasty (UKA), and total knee arthroplasty (TKA) are available [2–5, 7–10]. Open-wedge HTO (OWHTO) is a well-established procedure for the treatment of SONK in the femoral condyle, and good cartilage regeneration after OWHTO has been reported [11, 12]. However, to our knowledge, there have been no reports regarding the clinical outcomes of OWHTO for spontaneous osteonecrosis of the medial tibial plateau.

The purpose of this study was to assess the clinical outcomes of OWHTO for spontaneous osteonecrosis of the medial tibial plateau at a single center in Japan. We hypothesized that OWHTO is an effective treatment for spontaneous medial tibial osteonecrosis, similar to that observed with SONK in the femoral condyle.

## Materials and methods

This retrospective case series was approved by our Institutional Review Board (IRB No. 2019–14), and all patients provided informed consent prior to participating in the study. Patients who underwent OWHTO for spontaneous osteonecrosis of the medial tibial plateau from November 2013 to September 2017 at our institution and followed up for at least 2 years after surgery were included in this study. The diagnosis of tibial osteonecrosis was based on clinical findings and the results of radiographic and magnetic resonance imaging (MRI) analyses. Patients with history of alcohol abuse and corticosteroid therapy were excluded.

Our inclusion criteria for the OWHTO procedure were as follows: (1) symptomatic osteoarthritis (OA) and osteonecrosis of the medial compartment, (2) varus malalignment, which was defined as a femorotibial angle (FTA) > 176°, and (3) active patients who had good compliance with the postoperative rehabilitation program. There were no age restrictions. The contraindications for OWHTO were: (1) a history of joint infection, (2) symptomatic OA of the lateral compartment or patellofemoral joint, (3) joint instability, (4) FTA > 185°, (5) flexion contracture of > 15°, and (6) extensive collapse of the medial tibial plateau.

### Surgical procedure and postoperative rehabilitation

The surgical technique and preoperative planning used in the present study were those described previously [13]. The weight-bearing line was aimed at a point 65%–70% lateral to the transverse diameter of the tibial plateau. Arthroscopy was routinely conducted prior to OWHTO to evaluate the medial, lateral, and patellofemoral cartilage. Damaged cartilage tissue was removed arthroscopically, the osteonecrosis lesion was curetted, and microfracture of the necrotic area was then performed. The biplanar OWHTO was internally fixed with a TomoFix® plate (DePuy Synthes, Solothurn, Switzerland). No bone graft or bone substitute was placed in the osteotomy site. Isometric quadriceps, active ankle exercises, and straight leg raises were started on the first postoperative day. Partial weight-bearing started 1 week after surgery. Full weight-bearing was permitted after 4 weeks.

### Clinical evaluations

Clinical evaluations, including range of motion (ROM), the Japanese Orthopedic Association (JOA) score [14], and the 12-item Oxford Knee Score (OKS) [15], were measured prior to surgery and at the final follow-up by a physician independent of the surgical team who was blinded to the radiographic findings. The OKS includes 12 questions regarding pain and function that are answered according to one of five response categories. The final score ranges from 0 to 48 points, with 48 representing the best outcome. In addition, we evaluated postoperative complications that required additional surgery and patient subjective satisfaction at the final follow-up using a five-point scale (very satisfied, satisfied, neutral, dissatisfied, or very dissatisfied).

### Radiological evaluations

The radiographic staging of the tibial osteonecrosis was assessed according to Carpintero [16], Lotke [6], and the modified Ficat and Arlet classification [5, 9] for the knee (Table 1). The radiological outcomes, including FTA, weight-bearing line ratio (WBLR), posterior tibial slope (PTS), and medial proximal tibial angle (MPTA), were evaluated preoperatively and at the final follow-up [17]. PTS was measured using standing lateral radiographs, and the other radiological parameters were measured using the Antero-posterior (AP) long-standing view of the lower extremity, which was taken with the knee in full extension and the patella facing forward at shoulder width in a weight-bearing stance. FTA was defined as the lateral angle between the axis of the femoral shaft and the axis of the tibial shaft. To calculate the WBLR, a line was drawn from the center of the femoral head to the midpoint of the proximal talar joint surface. The WBLR was defined as the horizontal distance from the WBL to the medial edge of the tibial plateau divided by the width of the tibial plateau. The MPTA is the medial angle formed between the mechanical tibial axis and the joint line of the proximal tibia. PTS was defined as the angle between a line perpendicular to the mid-diaphysis of the tibia and the posterior inclination of the tibial plateau.

**Table 1:** Staging of osteonecrosis based on planar radiography and magnetic resonance imaging (MRI) according to Carpintero [16], Lotke [6], and the modified Ficat and Arlet classification [5, 9]

Stage	Radiography	MRI
1	Normal	Relatively small and well localized, low signal in subchondral zone (T1)
2	Abnormal, cystic, and sclerotic changes	Low signal area in subchondral zone diffused down to metaphysis
3	Crescent sign and subchondral collapse producing crescent or rim sign	Changes with widespread diffusion in metaphysis, surrounded by reactive bone rim
4	Arthritic changes joint narrowing with or without condylar involvement	Diffuse areas of abnormal marrow signal intensity, involvement of the condyle possible

### MRI evaluations

**Fig. 1:**
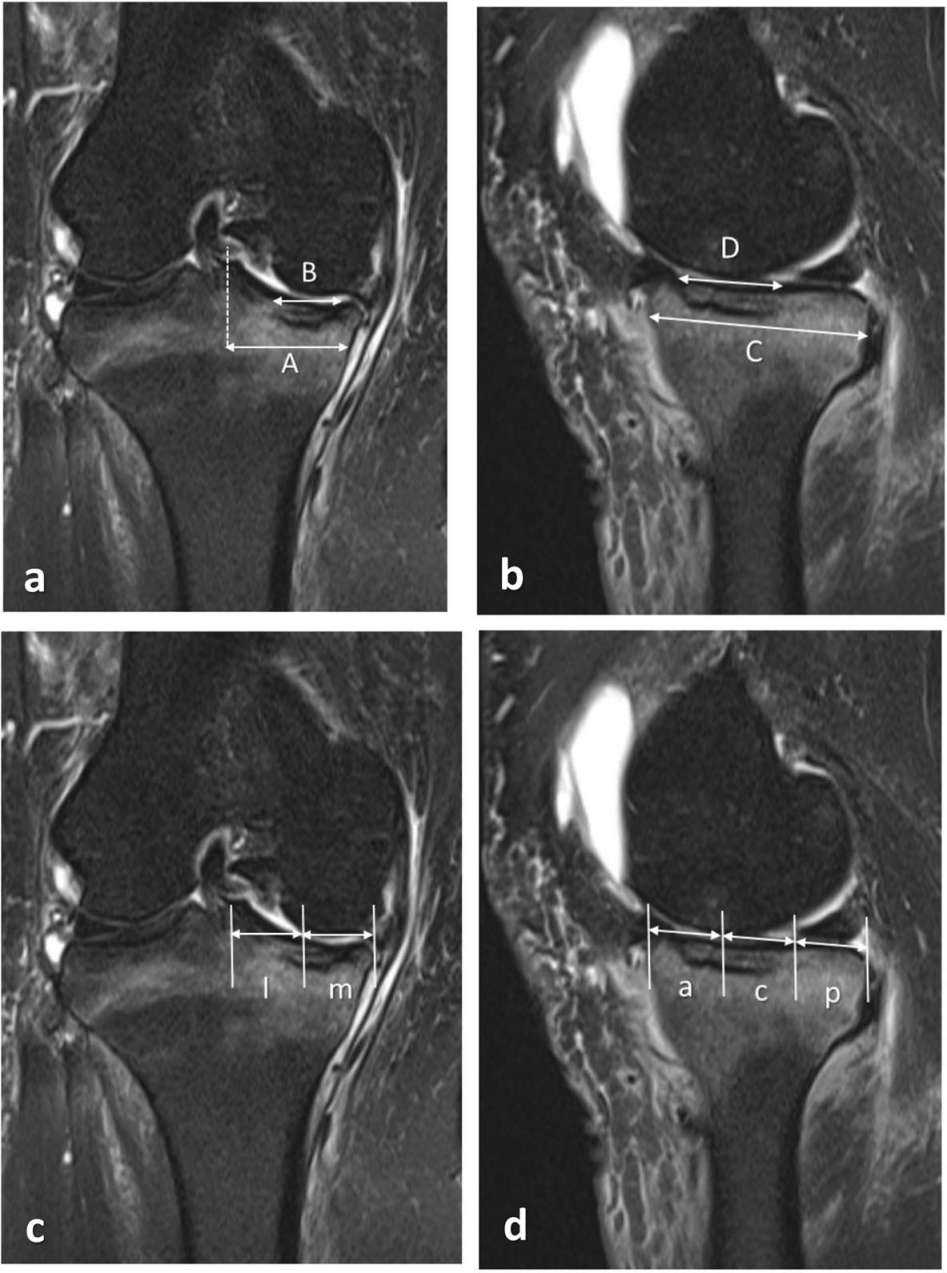
Evaluations of SONK lesion size and location using magnetic resonance imaging. **a, b** The size of the necrotic lesion was calculated as the percentage (anteroposterior (AP) = D/C, mediolateral (ML) = B/A) of the AP and ML width of the medial compartment in the sagittal (**b**) and coronal (**a**) planes. The ML width was defined as the distance between the medial intercondylar eminence and the edge of the medial tibial plateau. The AP width was measured on the sagittal plane passing through the center of the ML line. **c**, **d** The location of the necrotic lesion was divided into two by bisecting the ML lines in the coronal (**c**) planes. l, lateral; m, medial. In the sagittal (d) plane, the lesion area was also divided into equal anterior (**a**), central (**c**), and posterior (p) sections

The location and size of necrosis and the presence of any meniscal lesions were assessed by preoperative MRI. The size of necrosis was calculated as a percentage of the AP and mediolateral (ML) widths of the medial compartment, where the ML width was defined as the distance between the medial intercondylar eminence and the edge of the MTP; the AP width was measured on the sagittal plane passing through the center of the ML width (Fig. 1a, b). The location of necrosis was divided into equal anterior, central, and posterior sections on the sagittal plane. In the coronal plane, the lesion was divided into two areas (medial and lateral) by bisecting ML distances (Fig. 1c, d). Extrusion of the medial meniscus was assessed using the edge of the tibial plateau (excluding osteophytes) as the reference and was assigned grade 0–2 (grade 0, no extrusion; grade 1, extrusion ≤50% of the body; grade 2, extrusion > 50% of the body as reported by Crema MD et al. [6]).

### Arthroscopic evaluations

Second-look arthroscopy was performed in 11 patients at the time of plate removal (postoperative 14.0 ± 3.5 months). The grade of degeneration-associated cartilage injury was assessed in accordance with the International Cartilage Repair Society classification system [16]. Cartilage regeneration in the medial compartment was classified into regeneration and no regeneration groups according to Jung et al. [18].

### Statistical analysis

JMP version 11 (SAS Institute Inc., Cary, NC) was used to analyze and manage the data. The data are presented as the means and standard deviations. The Student’s *t-*test was used to analyze the quantitative data. *P <* 0.05 was considered statistically significant. All of the radiological parameters were measured twice by two observers with an interval of more than 4 weeks between each measurement. The observers were blinded to the previous observations. The reliability of measurements was assessed by examining the intra-rater and inter-rater reliabilities using the intra-class correlation coefficient. The intra- and inter-observer reliabilities for the measurement of radiological parameters were satisfactory, and the mean values were 0.95 (range, 0.73 to 0.99) and 0.91 (range, 0.78 to 0.99), respectively.

## Results

**Fig. 2:**
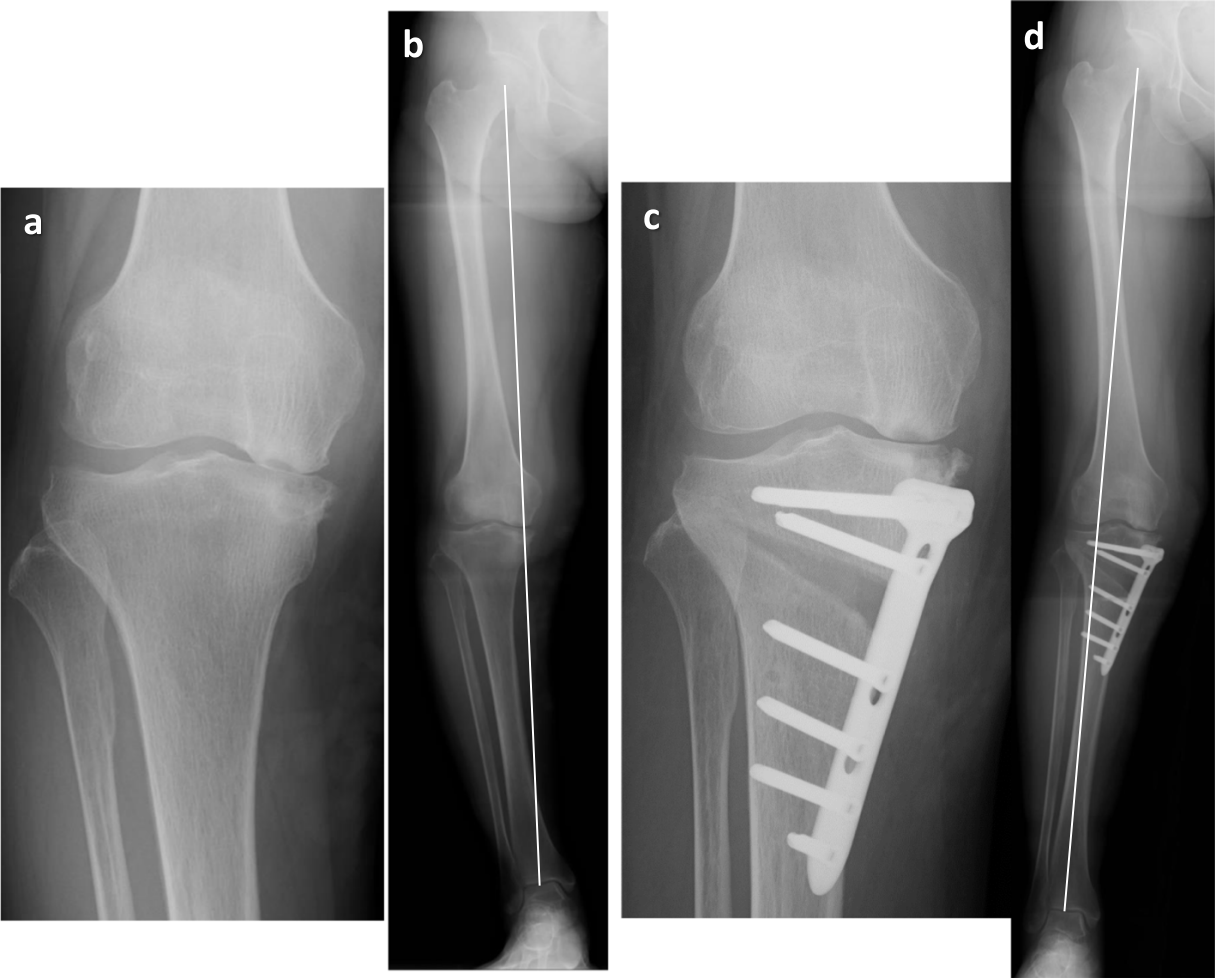
A case of a 58-year-old woman with both femoral and tibial osteonecrosis. **a** Antero-posterior (AP) standing radiograph of the knee prior to surgery. Collapse of the medial tibial plateau and joint space narrowing was observed (Stage 4). **b** AP standing radiograph of the entire leg prior to surgery. The weight-bearing line ratio (WBLR) was 10.7%. The Oxford knee score was 30 points. **c** AP standing radiograph after OWHTO. **d** AP standing radiograph of the entire leg after OWHTO. The WBLR was 62.7%

Twelve patients who underwent OWHTO for spontaneous medial tibial osteonecrosis from November 2013 to September 2017 at our institution were included in this study. Eleven cases had isolated medial tibial osteonecrosis, and one case had both femoral and tibial osteonecrosis (Fig. 2). All patients had no history of alcohol abuse and had not received corticosteroid therapy. The mean age at the time of surgery was 59.6 ± 9.0 years (range, 44–75 years), and the mean body mass index was 24.6 ± 3.8 kg/m^2^ (range, 20.1–34.6 kg/m^2^). The mean follow-up period was 41.8 ± 17.6 months (range, 25.1–71.1 months). The patient characteristics are shown in Table 2.

**Table 2:** Clinical data of the patients

Patient number	Age (years)	Sex (F/M)	BMI (kg/m^2^)	DEXA t value	Interval symptom to ON (weeks)	Stage	FTA (°)	Follow-up (Months)
1	56	F	24.2	−0.6	8	4	180.6	71.1
2	58	F	22.1	−3.7	20	4	182	70.5
3	67	F	22.9	0.4	20	3	184	61.2
4	54	F	28.8	−1.2	28	3	177	52.5
5	60	M	23.6	−0.4	12	3	179.3	42.4
6	66	F	24.0	2.4	4	4	179	37.0
7	67	M	20.1	−0.7	12	3	177	31.4
8	66	F	23.6	−1.2	6	4	179	29.5
9	75	F	22.6	−3.1	3	3	177.1	28.1
10	44	M	34.6	−0.3	20	4	182	27.1
11	55	M	26.3	−1.9	12	4	178	25.7
12	57	M	22.2	−0.3	16	4	178	25.1

Mean (standard deviation, range)

Abbreviations: *F/M* Female/male, *BMI* Body mass index, *DEXA* Dual energy X-ray absorptiometry, *ON* Osteonecrosis, *FTA* Femorotibial angle

Staging of tibial osteonecrosis according to Carpintero, Lotke, and the modified Ficat and Arlet classification

### Clinical outcomes

The results of the clinical evaluations are shown in Table 3. ROM and clinical scores significantly improved after OWHTO. The mean OKS significantly improved from the preoperative value of 27.4 ± 7.8 points (range, 15–37) to 42.6 ± 4.1 points (range, 36–48) at the final follow-up (*P <* 0.001). Regarding the postoperative complications, there were no adverse events requiring additional surgery such as infection, non-union, and implant failure. All patients were satisfied with their surgery (very satisfied in nine knees and satisfied in three knees).

**Table 3:** Preoperative and postoperative comparison of clinical and radiological outcomes

	Pre-operation	Post-operation	*P* value
Ext. angle (°)	−4.2 (4.2, −10.0–0.0)	−0.8 (2.0, −5.0–0.0)	0.0201
Flex. angle (°)	134.2 (4.7, 125.0–140.0)	141.3 (5.3, 135.0–150.0)	0.0021
JOA score (points)	63.3 (12.3, 40.0–85.0)	95.0 (4.8, 85.0–100.0)	< 0.001
OKS (points)	27.4 (7.8, 15.0–37.0)	42.6 (4.1, 36.0–48.0)	< 0.001
FTA (°)	179.3 (2.3, 177.0–184.0)	169.1 (1.5, 166.2–172.0)	< 0.001
WBLR (%)	24.0 (10.7, 0.6–37.9)	66.3 (6.7, 58.0–77.6)	< 0.001
PTS (°)	9.0 (1.7, 7.1–13.0)	9.1 (1.9, 6.6–13.3)	n.s.
MPTA (°)	83.1 (1.7, 81.2–85.0)	92.6 (2.1, 89.7–94.6)	< 0.001

Mean (standard deviation, range)

Abbreviations: *n.s.* Non-significant, *JOA* Japanese Orthopedic Association, *OKS* Oxford knee score, *FTA* Femorotibial angle, *WBLR* Weight-bearing line ratio, *PTS* Posterior tibial slope, *MPTA* Medial proximal tibial angle

### Radiological outcomes

The lesion stage assessment showed that seven patients were stage 4 and five patients were stage 3. The results of the radiological evaluations are listed in Table 3. The mean opening width was 11.5 ± 2.1 mm (range, 8.0–14.0 mm). The mean FTA significantly improved from 179.3 ± 2.3° (range, 177.0°–184.0°) preoperatively to 169.1 ± 1.5° (range, 166.2°–172.0°) at the final follow-up. The postoperative WBLR was 66.3 ± 6.7% (range, 58.0%–77.6%).

### MRI study

The MRI findings are shown in Table 4. In the coronal plane, all cases were in the medial region. In the sagittal plane, five cases were in the anterior and central region, four were in the anterior region, and three were in the central region. The mean percentage of necrosis was 39.9 ± 8.1% (range, 26.6%–53.3%) in the AP direction and 43.8 ± 8.3% (range, 31.9%–57.9%) in the ML direction. Nine patients (75.0%) had a posterior root tear, and three patients had a horizontal tear; a medial meniscal extrusion was observed in all patients.

**Table 4:** Magnetic resonance imaging (MRI) findings of the patients

Patient number	Stage in MRI	Necrotic area		Necrosis size (%)		Medial meniscal tear	Meniscal extrusion grade
		AP	ML	AP	ML		
1	4	Ant	Med	29.4	38.9	Root tear	2
2	4	Ant/central	Med	45.8	43.5	Root tear	2
3	3	Central	Med	37.3	49.4	Horizontal tear	2
4	3	Ant/central	Med	43	31.9	Root tear	2
5	3	Ant	Med	26.6	33.9	Root tear	1
6	4	Ant/central	Med	53.3	57.9	Root tear	2
7	3	Central	Med	43.1	37.6	Root tear	2
8	4	Ant	Med	39.8	41.5	Root tear	2
9	3	Ant	Med	31	42.1	Root tear	2
10	4	Ant/central	Med	39.9	47.2	Root tear	2
11	4	Ant/central	Med	50.2	57.9	Horizontal tear	1
12	4	Central	Med	38.9	43.5	Horizontal tear	2

Abbreviations: *AP* Anteroposterior, *ML* Mediolateral, *Ant* Anterior, *Med* Medial

### Arthroscopic findings

**Fig. 3:**
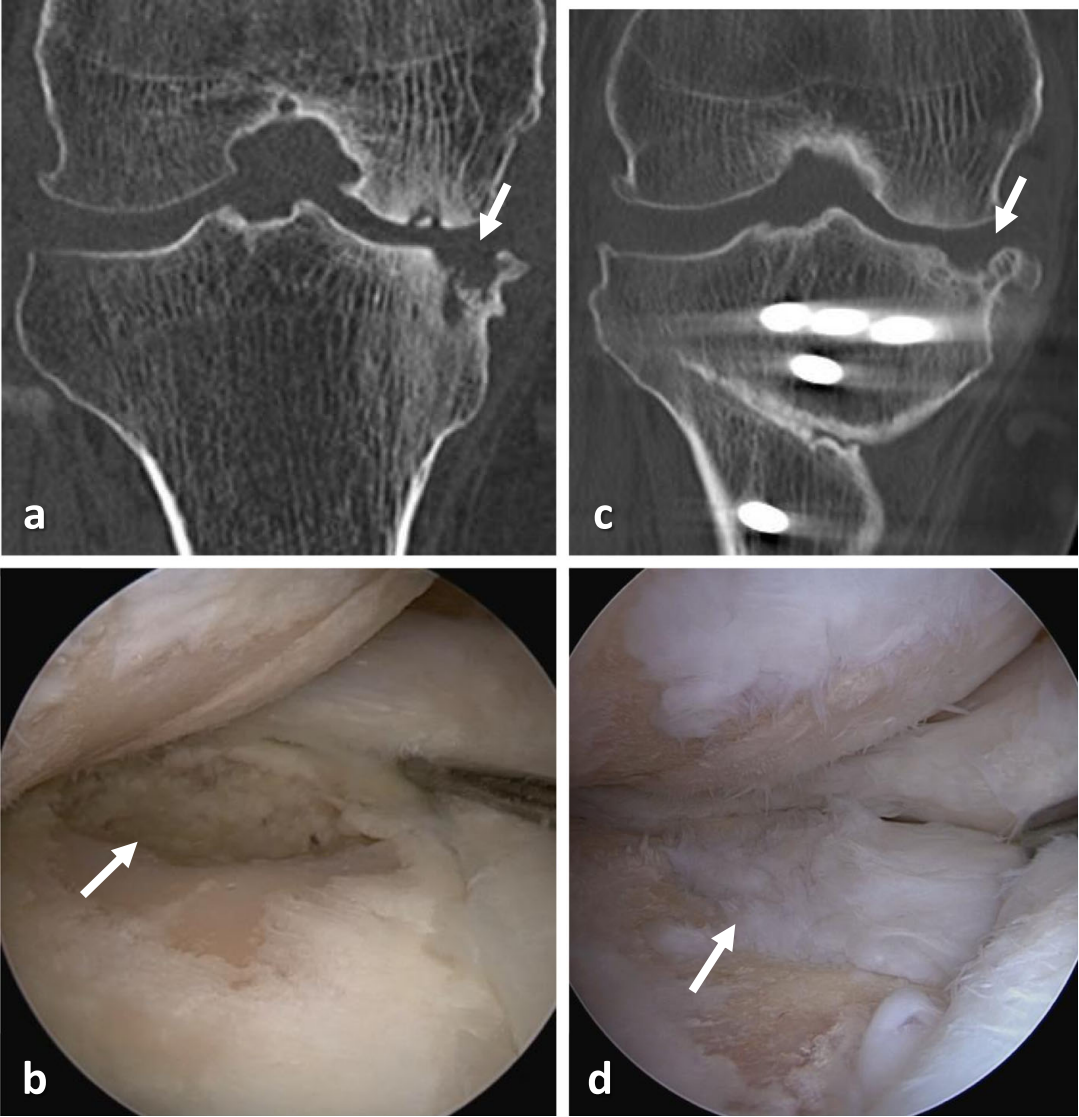
CT and arthroscopic findings of the case shown in Fig. 2. **a** CT findings before OWHTO. The subchondral collapse of the medial tibial plateau was observed. **b** Arthroscopic findings during OWHTO showing the subchondral collapse and the defect of the medial tibial plateau. **c** CT findings at 19 months after OWHTO (at plate removal). The necrotic lesion has decreased in size, and remodeling of the lesion was observed. **d** Arthroscopic findings at plate removal. The necrotic lesion of the medial tibial plateau was covered with fibrous cartilage-like tissue

Second-look arthroscopy was performed at plate removal in 11 cases. The mean postoperative time until second-look arthroscopy was 14.0 ± 3.5 months (range, 9.8–21.2 months). Cartilage regeneration was observed in 9/11 cases (81.8%), and the necrotic area was covered with fibrous cartilage-like tissue (Fig. 3).

## Discussion

The principal finding of this study was that OWHTO is an effective treatment for spontaneous osteonecrosis of the medial tibial plateau, similar to that for SONK in the femoral condyle. In addition, patient subjective satisfaction after OWHTO was high, and the necrotic lesion was covered with fibrous cartilage-like tissue arthroscopically without requiring any cartilage repair procedures in most cases.

Although the etiology of SONK remains unclear, two classical theories have been suggested: primary vascular insufficiency leads to infarction of the bone [19], or trauma with microfractures and subsequent osteonecrosis [20]. In most cases, minor trauma or mechanical stress causes defects in the subchondral bone plate and leads to subchondral microfracture and vascular disturbance of the bone beneath, resulting in osteonecrosis [21, 22]. In addition to osteoporosis [23], meniscal function insufficiency including posterior root tears and meniscal extrusion are identified as highly correlated with SONK in the femoral condyle [24, 25]. Robertson et al. [26] reported that posterior medial meniscus root tears (MMRTs) were found in 80% of patients with SONK in the femoral condyle and assumed that the mechanical overload caused by an MMRT would play a role in the development of SONK. In addition, Kamenaga et al. [7] reported a case series of six patients with osteonecrosis of the medial tibial plateau, and all cases had medial meniscal tears and meniscal extrusion, which coincided with the present study. Although direct correlation between meniscus injury and osteonecrosis of the tibial plateau cannot be proven by retrospective small case series, these meniscus lesions may be associated with the occurrence of SONK in the tibial plateau.

Stage-dependent treatment is recommended for osteonecrosis of the medial tibial plateau [5, 7–9]. In the early stages, conservative treatment, including brace application and restrictions in weight bearing, may be successful depending on the size of the defects and the integrity of the cortical rim. However, the natural course of tibial plateau osteonecrosis may differ from SONK in the femoral condyle. Satku et al. [21] investigated the natural history of spontaneous osteonecrosis of the medial tibial plateau and concluded that the affected lesion progresses in most cases to significant degenerative disease of the knee. Unlike the femoral side where the contact point changes according to the knee flexion angle, the weight load is concentrated within a limited area on the tibial plateau during knee motion. Therefore, conservative treatment for tibial plateau osteonecrosis may be less successful compared to that in the femoral side. A close follow-up is necessary in conservative treatment for osteonecrosis of the tibial plateau.

In the advanced stage, surgical treatments such as HTO, UKA, and TKA can be selected according to the patient’s age, activity, ROM, lower limb alignment, and degree of collapse. UKA is an effective treatment method for osteonecrosis of the medial tibial plateau, with the benefit of preserving the patient’s bone stock and functioning cruciate ligaments. Kamenaga et al. [7] reported a good short-term clinical outcome of Oxford mobile-bearing UKA for tibial plateau osteonecrosis. However, the aseptic loosening of the tibial component is a concern in this type of osteonecrosis because good bone quality is essential for reliable fixation of the tibial component [27]. When considering the size of the necrotic area, TKA would be a safer choice for larger areas rather than UKA [3, 10].

**Fig. 4:**
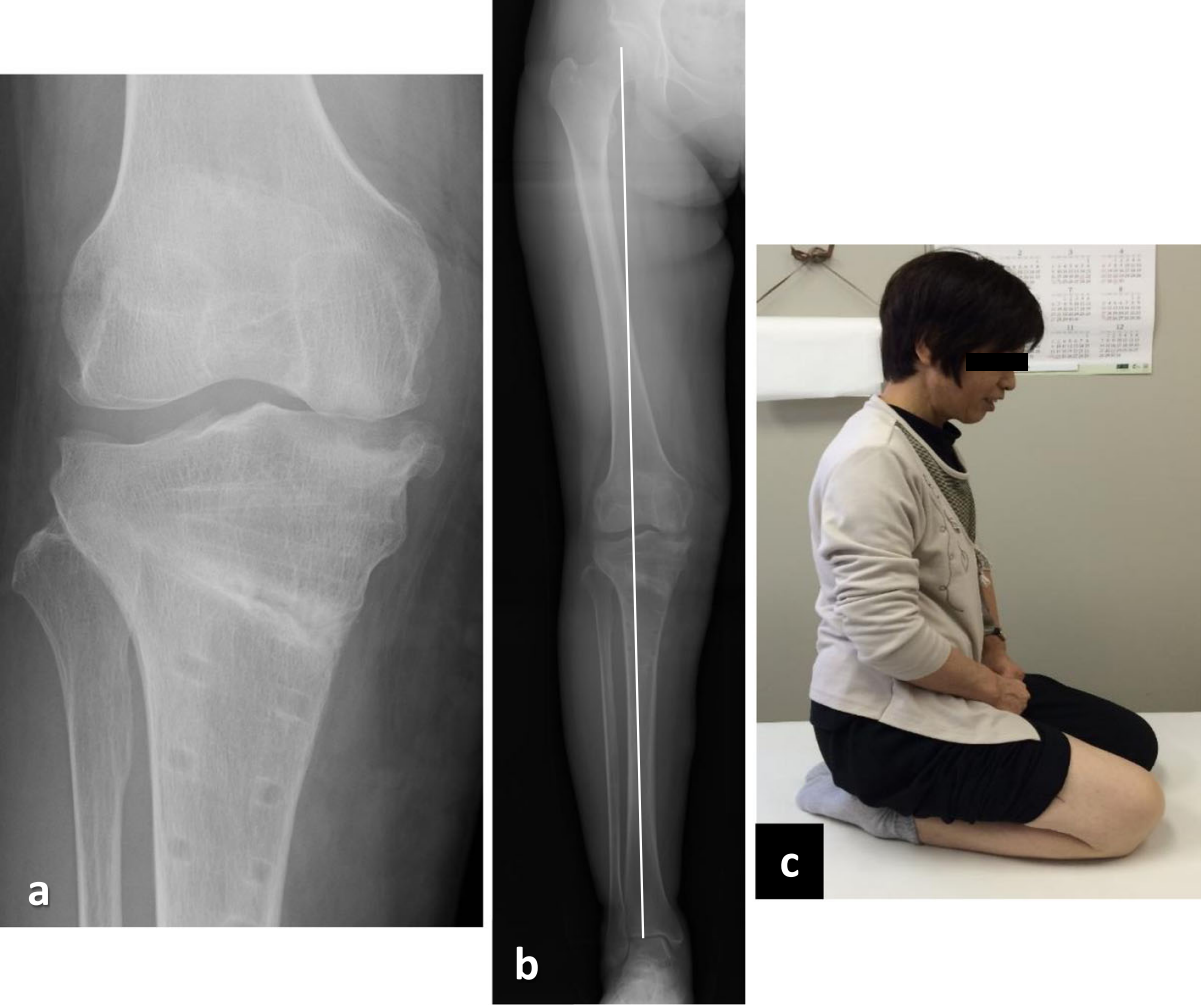
The case at last follow-up (three years after OWHTO). **a** The AP standing radiograph of the knee. **b** The AP standing radiograph of the whole leg. The WBLR was 61.5%, and the lower leg alignment was maintained. **c** The patient could sit Japanese style. The Oxford knee score at last follow-up was 43 points

OWHTO with the rigid locking plate is a well-established procedure for the treatment of SONK in the femoral condyle, and good cartilage regeneration after OWHTO has been reported [11, 12]. However, there have been no reports regarding the clinical outcomes of OWHTO for osteonecrosis of the tibial side. The present study demonstrated a good clinical outcome of OWHTO for this type of osteonecrosis. In addition, good patient satisfaction and cartilage regeneration were observed in this study (Figs. 3 and 4). Considering that one of the main causes of SONK is mechanical stress due to meniscal function insufficiency including posterior root tears and meniscal extrusion, it is theoretical to improve the biomechanical status of the osteonecrosis lesion by the unloading effects of HTO [28]. However, in cases of extensive collapse of the tibial plateau, TKA must be selected [10]. Satku et al. [21] reported the natural course of 21 cases with tibial osteonecrosis; two patients experienced acute extensive collapse within 3 months of onset and three experienced had rapid progression to severe osteoarthritis requiring TKA. Therefore, it is critical to perform HTO before the lesions progress extensively.

### Limitations

There were several limitations in this study. First, we retrospectively analyzed the clinical outcomes of OWHTO in tibial plateau osteonecrosis without a control group undergoing another treatment. Second, a small number of patients were included in this study. Although osteonecrosis of the medial tibial plateau is a rare condition compared to osteonecrosis of the femoral condyle, the small sample size was a significant and major limitation of this study. Lastly, the follow-up period was relatively short. Further studies with a larger sample size and long-term follow-up are necessary. Despite these limitations, this is the first reported study that investigated the clinical outcomes of OWHTO for spontaneous osteonecrosis of the medial tibial plateau.

## Conclusions

The results of this study demonstrated that OWHTO is an effective procedure for spontaneous osteonecrosis of the medial tibial plateau with respect to subjective and objective clinical outcomes.
